# Gilteritinib: a novel FLT3 inhibitor for acute myeloid leukemia

**DOI:** 10.1186/s40364-019-0170-2

**Published:** 2019-09-11

**Authors:** Juanjuan Zhao, Yongping Song, Delong Liu

**Affiliations:** 1 Department of Oncology, The first Affiliated Hospital of Zhengzhou University, Zhengzhou, 450052 China; 2 Division of Hematology & Oncology, New York Medical College, Valhalla, NY 10595 USA

**Keywords:** FLT3, Gilteritinib, Tyrosine kinase inhibitor, FLT3 inhibitor

## Abstract

FMS-like tyrosine kinase 3- internal tandem duplication (FLT3-ITD) remains as one of the most frequently mutated genes in acute myeloid leukemia (AML), especially in those with normal cytogenetics. The FLT3-ITD and FLT3-TKD (tyrosine kinase domain) mutations are biomarkers for high risk AML and are associated with drug resistance and high risk of relapse. Multiple FLT3 inhibitors are in clinical development, including lestaurtinib, tandutinib, quizartinib, midostaurin, gilteritinib, and crenolanib. Midostaurin and gilteritinib have been approved by FDA for Flt3 mutated AML. Gilteritinib (ASP2215, Xospata) is a small molecule dual inhibitor of FLT3/AXL. The ADMIRAL study showed that longer overall survival and higher response rate are associated with gilteritinib in comparison with salvage chemotherapy for relapse /refractory (R/R) AML. These data from the ADMIRAL study may lead to the therapy paradigm shift and establish gilteritinib as the new standard therapy for R/R FLT3-mutated AML. Currently, multiple clinical trials are ongoing to evaluate the combination of gilteritinib with other agents and regimens. This study summarized clinical trials of gilteritinib for AML.

## Background

Recurrent and novel genetic mutations are increasingly discovered through FISH, PCR and next-generation sequencing studies of leukemia specimens [1–5]. These findings led to new classifications of leukemia [2, 6]. New agents targeting these recurrent mutations are rapidly emerging for high-risk acute myeloid leukemia (AML) [7, 8]. Among these common mutations, FMS-like tyrosine kinase 3-internal tandem duplication (FLT3-ITD) remains as one of the most frequently mutated genes in AML, especially in those with normal cytogenetics, in which the mutation rate can be as high as 30% [9–11].

FLT3 gene encodes a receptor type tyrosine kinase which plays a key role in the proliferation, differentiation, and survival of hematopoietic stem cells. FLT3-ITD leads to constitutive activation of the FLT3 tyrosine kinase, resulting in uncontrolled cell proliferation and high WBC counts in AML patients [12, 13].

The FLT3-ITD and FLT3-TKD (tyrosine kinase domain) mutations are biomarkers for high risk AML and are associated with drug resistance and high risk of relapse [14, 15], particularly in those patients with wild-type NPM1 and high alleilic ratio of FLT3-ITD. These mutations can also serve as biomarkers for minimal residual diseases [16]. Allogeneic hematopoietic stem cell transplantation (HSCT) is routinely recommended for AML patients with high alleilic ratio of FLT3/ITD and TKD mutations [17]. Oral tyrosine kinase inhibitors (TKI) are widely used for targeted therapy of chronic myeloid leukemia and myeloproliferative neoplasms [18–21]. FLT3/ITD and FLT3/TKD are ideal targets for small molecule inhibitors. Multiple FLT3 inhibitors are in clinical development, including sorafenib, lestaurtinib, sunitinib, tandutinib, quizartinib, midostaurin, gilteritinib, crenolanib, cabozantinib, Sel24-B489, G-749, AMG 925, TTT-3002, and FF-10101 [22–30]. Midostaurin and gilteritinib have been approved by FDA for Flt3 mutated AML [31]. This study summarized clinical trials of gilteritinib for AML.

### Gilteritinib (ASP2215, Xospata) for relapsed and /or refractory AML (R/R AML)

Gilteritinib is a small molecule dual inhibitor of FLT3/AXL. In a phase I/II study in relapsed /refractory (R/R) AML with or without FLT3 mutations, gilteritinib was given as once-daily doses in dose-escalation and dose-expansion cohorts (20 mg, 40 mg, 80 mg, 120 mg, 200 mg, 300 mg, or 450 mg) (Table 1, NCT02014558). In the expansion cohort, doses at 120 mg and 200 mg were given to those R/R AML with FLT3 mutations. In the published report, 23 patients were enrolled in the dose-escalation cohort, 229 patients were included in the dose-expansion cohort [32]. The dose-limiting toxicities (DLT) were grade 3 diarrhea and elevated aspartate aminotransferase (ALT) at the daily dose of 450 mg. Therefore 300 mg/day was the maximum tolerated dose (MTD). The most common treatment-emergent adverse events (TEAE) were diarrhea, anemia, fatigue, and liver enzyme elevation. In the group of 249 patients for full analysis, overall response rate (ORR) was 40%. In summary, gilteritinib was well tolerated in patients with R/R AML. This trial established the daily dose of 120 mg gilteritinib for further clinical phase 3 trials.

**Table 1:** Clinical trials of gilteritinib for acute myeloid leukemia

No.	AML status	therapy	Phase	NCT	Trial Status
1	R/R AML	Gilteritinib	Phase 1	02181660	Completed
2	R/R AML	Gilteritinib	Phase 1/2	02014558	Completed
3	Previously Untreated AML with FLT3 Mutation	Gilteritinib	Phase 1/2	03013998	Recruiting
4	Advanced Solid Tumors and AML	Gilteritinib	Phase 1/2	02561455	Enrolling by invitation
5	R/R AML with FLT3 Mutation or AML with FLT3 Mutation in CR with MRD	Gilteritinib	NA	03070093	Available
6	R/R AML with FLT3 Mutation or AML with FLT3 Mutation in CR with MRD	Gilteritinib	NA	03409081	No longer available
7	Pediatric R/R AML with FLT3 Mutation or AML with FLT3 Mutation in CR with MRD	Gilteritinib	NA	03315299	No longer available
8	R/R AML	Gilteritinib + Venetoclax	Phase 1	03625505	Recruiting
9	Newly Diagnosed AML	Gilteritinib + Cytarabine + Idarubicin	Phase 1	02310321	Active, not recruiting
10	Newly Diagnosed AML	Gilteritinib + Cytarabine + Idarubicin or Gilteritinib + Cytarabine + Daunorubicin	Phase 1	02236013	Recruiting
11	R/R AML with FLT3 Mutation	Gilteritinib + Atezolizumab	Phase 1/2	03730012	Recruiting
12	AML with FLT3/ITD Mutation in CR1	Gilteritinib vs Placebo	Phase 2	02927262	Active, not recruiting
13	Untreated AML with FLT3 Mutation	Gilteritinib + Daunorubicin + Cytarabine vs Midostaurin + Daunorubicin + Cytarabine	Phase 2	03836209	Not yet recruiting
14	Newly Diagnosed AML With FLT3 Mutation	Gilteritinib vs Gilteritinib + Azacitidine vs Azacitidine	Phase 2/3	02752035	Recruiting
15	AML With FLT3/ITD Mutation in CR1 undergoing allo-HSCT	Gilteritinib vs Placebo	Phase 3	02997202	Recruiting
16	R/R AML with FLT3 Mutation	Gilteritinib vs Salvage Chemotherapy	Phase 3	03182244	Recruiting
17	R/R AML with FLT3 Mutation	Gilteritinib vs Salvage Chemotherapy	Phase 3	02421939	Active, not recruiting
18	Newly Diagnosed AML or MDS-EB2 with FLT3 mutation	Gilteritinib vs Midostaurin in Combination With chemotherapy	Phase 3	04027309	Not yet recruiting

Abbreviations: *R/R*, Relapsed or Refractory; *FLT3*, FMS-like Tyrosine Kinase 3; *ITD*, Internal Tandem Duplication; *CR*, Complete Remission; *MRD*, Minimal Residual Disease; *MDS-EB2*, Myelodysplastic Syndromes with Excess Blasts-2; *NA*, Not Available; *allo-HSCT*, allogeneic Hematopoietic Stem Cell Transplant

Gilteritinib was studied in another phase 1 study in Japanese patients with R/R AML (table, NCT02181660). Gilteritinib was given as daily escalating doses in 6 cohorts, with doses ranging from 20, 40, 80, 120, 200, to 300 mg/day. In the published report, 24 subjects were enrolled [33]. Grade 3 tumor lysis syndrome (TLS) was observed at the dose 120 mg/day in one patient. At 300 mg/day, two patients developed grade 3 elevated lactate dehydrogenase (LDH), amylase, creatine phosphokinase levels, and syncope. These grade 3 toxicities were DLTs. The MTD was established at 200 mg/day. Among the 5 patients with FLT3 mutations, the ORR was 80% (*n* = 4). Four of 11 patients with wild-type FLT3 also responded. This study also established the 120 mg once-daily as the recommended dose in the Japanese patients.

**Fig. 1:**
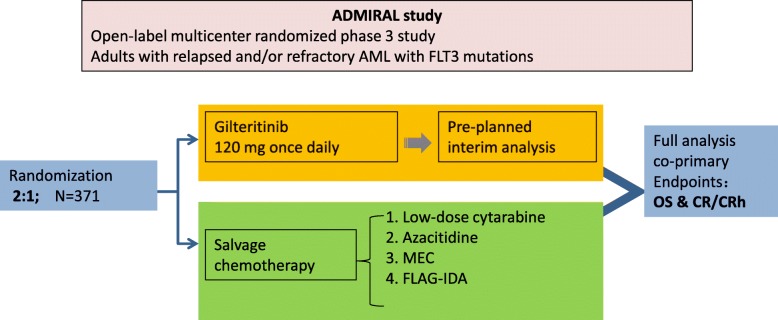
The ADMIRAL study scheme. The ADMIRAL study is an open-label multicenter randomized phase 3 study for adults with relapsed and/or refractory AML with FLT3 mutations. The randomization between gilteritinib and one of the four salvage chemotherapy regimens was 2:1. To date, the accrual has been completed with 371 patients randomized. MEC: mitoxantrone, etoposide, cyclophosphamide; FLAG-IDA: fludarabine, cytarabine, GCSF, idarubicin; CR/CRh: complete remission with partial hematologic recovery; OS: overall survival

Gilteritinib was compared with salvage chemotherapy in R/R AML with mutated FLT3 in an open-label, multicenter, randomized phase III study (ADMIRAL study; NCT03182244) [34]. Gilteritinib was given at 120 mg daily, and randomized as 2:1 with one of the four salvage chemotherapy regimens (low-dose cytarabine, azacitidine, MEC, or FLAG-IDA) (Fig. 1). The primary endpoints were OS and CR/CRh (complete remission with partial hematologic recovery). The accrual has been completed with 371 patients randomized.

In the pre-planned interim analysis of 138 patients enrolled in the gilteritinib group, CR/CRh was 21% (95% CI 14.5–28.8, *n* = 29/138), with 11.6% CR and 9.4% CRh. The median duration of remission (DOR) was 4.6 months (range 0.1–15.8). The median time to response was 3.6 months (range 0.9–9.6). Based on these data, gilteritinib was approved by US FDA for R/R AML patients [35].

Among the 371 patients, 247 were randomized to gilteritinib and 124 to salvage chemotherapy [34]. The median age of the 371 patients was 62 years (range 19–85). Among the FLT3 mutations, 88.4% were FLT3-ITD, 8.4% were FLT3-TKD, and 1.9% were both FLT3-ITD and FLT3-TKD, 1.3% had unconfirmed mutations. The OS was significantly longer in the gilteritinib group (9.3 months) than that in the SC group (5.6 months) (hazard ratio [HR] for death = 0.637; *P* = 0.0007). The rates of OS at 1 year were 37.1% for gilteritinib group and 16.7% for the SC group. In the full analysis of the 371 patients randomized, the CR/CRh rate for gilteritinib group (34%) was significantly better than that in the SC group (15.3%, *P* = 0.0001). Cytopenia were the common serious adverse events (SAE) related to gilteritinib, including anemia, febrile neutropenia, and thrombocytopenia. Other clinically significant AEs that were observed in the clinical trials of gilteritinib included prolonged cardiac ventricular repolarization (QT interval, QTc, 9%), pancreatitis (5%), posterior reversible encephalopathy syndrome (1%), and differentiation syndrome (3%). Dexamethasone 10 mg IV every 12 h (or an equivalent dose of an alternative oral or IV corticosteroid) should be initiated once differentiation syndrome is suspected. Careful hemodynamic monitoring should be done until clinical improvement. Steroids should be administered for a minimum of 3 days and can be tapered once the symptoms resolve.

In conclusion, gilteritinib as a single oral agent at 120 mg daily led to significantly longer OS and higher ORR than salvage chemotherapy. The overall safety profile also favors gilteritinib. These data from the ADMIRAL study may lead to the therapy paradigm shift and establish gilteritinib as the new standard therapy for R/R FLT3-mutated AML.

### Gilteritinib in combination regimens

The role of gilteritinib in combination regimens remains unclear. It is possible that adding gilteritinib to the commonly used chemotherapy agents and regimens may improve clinical efficacies. Currently, multiple clinical trials are ongoing to evaluate the combination of gilteritinib with other agents and regimens (Table 1).

Epigenetic dysregulation plays a major role in leukemogenesis [36–39]. Hypomethylating agents have been shown to be active in AML as a single agent as well as in combination regimens [40–46]. A multicenter, open-label, 3-arm study is being done to compare gilteritinib, gilteritinib plus azacitidine (AZA), or azacitidine alone in newly diagnosed FLT3 mutated (FLT3 mut+) AML patients who are unfit for intensive induction chemotherapy (NCT02752035). To evaluate the appropriate gilteritinib dose for combination therapy prior to the 3-arm randomized phase, patients were enrolled in a safety cohort who received gilteritinib either 80 mg or 120 mg/day with AZA at 75 mg/m2 on days 1–7. Each treatment cycle is 28 days. In a recent update at the 2018 ASH annual meeting, 15 adult patients were recruited to the safety cohort [47]. Among these patients with a median age of 76 (range 65–86), 9 received gilteritinib at 80 mg, and 6 at 120 mg daily. One patient who received 80 mg gilteritinib plus AZA developed TLS as the DLT, whereas no DLTs were observed in patients who had 120 mg gilteritinib plus AZA. Cytopenia were the common SAEs. Eight patients had fatal events that were not related to therapy. The ORR was 80%, and composite CR was 67% (*n* = 10/15). In conclusion, the combination of gilteritinib with AZA was well tolerated. These data from the safety cohort led to the decision to use a dose of 120 mg gilteritinib plus AZA in the randomized portion of the 3-arm study. The preliminary data showed antileukemic responses in these newly diagnosed FLT3mut + elderly unfit AML patients. This trial could provide evidence for a new regimen for elderly AML patients [48].

In an ongoing open-label, dose-escalation /expansion phase 1 study, gilteritinib is being studied in combination with front-line 7 + 3 induction chemotherapy in adult patients with newly diagnosed AML (NCT02236013). This study also includes consolidation phase with high-dose cytarabine, and maintenance therapy with single-agent gilteritinib. Dose escalation of gilteritinib was planned at 40, 80, 120, or 200 mg/day. During the initial 2 cycles of a standard 7 + 3 induction regimen (cytarabine plus idarubicin [dose-escalation and dose-expansion cohorts], gilteritinib was given on days 4–17 [Schedule 1]). Once the dose-expansion cohort using Schedule 1 was completed, a new cohort of six patients were enrolled, with gilteritinib given on days 8–21 (Schedule 2). For the consolidation phase, cytarabine was planned at 1.5 g/m2 every 12 h on days 1, 3, and 5 together with gilteritinib on days 1–14. For responding subjects with appropriate donors, HSCT was allowed. For the maintenance phase after consolidation or transplantation, gilteritinib was given daily as a single agent for ≤26 cycles. In the update at the 2018 ASH annual meeting, 62 patients were enrolled, with 60 eligible for safety analysis [49]. Among these patients, FLT3 mutations were seen in 53.3%. DLTs with neutropenia, thrombocytopenia, and decreased ejection fraction were observed during the dose escalation at the dose 40 mg/day of gilteritinib. To reduce toxicities, the gilteritinib induction schedule was modified. After this adjustment, two patients reported DLTs with neutropenia and neutropenic enterocolitis in the 200 mg/day cohort. The MTD was established at 120 mg/day which was also recommended as the expansion dose. For FLT3mut + patients receiving gilteritinib 120 mg on Schedule 1, all 17 patients who were evaluable for efficacy achieved 100% composite CR (CRc). Interestingly, those patients receiving Schedule 2 induction with daunorubicin also had 100% CRc rate. Enrollment in the Schedule 2 cohort receiving idarubicin is ongoing; the two subjects in this cohort have not been assessed for response. Among 47 patients who received ≥80 mg/day gilteritinib, the CRc rate for those patients with FLT3 mutations reached 88.9% (*n* = 24/27). In conclusion, gilteritinib in combination with intensive chemotherapy for induction was well tolerated. Two different gilteritinib schedules in combination with idarubicin or daunorubicin induced high ORR in those patients with FLT3 mutations. Overall survival and long-term outcome are still being monitored.

Midostaurin has been approved for combination with induction chemotherapy for newly diagnosed AML patients with FLT3 mutations [30, 31, 50, 51]. A phase 3 randomized study has been planned to compare gilteritinib with midostaurin in combination with induction chemotherapy (NCT04027309, Table 1). Gilteritinib is also being studied in combination with venetoclax as a chemotherapy-free regimen for R/R AML (NCT03625505). More studies are being planned or ongoing for AML with FLT3 mutations (Table 1).

### Future perspectives

Among the FLT3 inhibitors in clinical trials, crenolanib is in multiple trials for R/R AML as well as for frontline regimens for newly diagnosed AML [22, 52, 53]. Patients who were resistant to gilteritinib and other FLT3 inhibitors were also being included in some studies of crenolanib [53, 54]. It is forseeable that more FLT3 inhibitors may become available for clinical applications [55–58]. It will be possible to choose among the approved agents according to a unique property for a particular patient in the near future. At this time, gilteritinib is the only approved FLT3 inhibitor as a single agent for R/R AML with FLT3 mutations, as suggested in the NCCN guidelines [59, 60].

Sorafenib, midostaurin as well as gilteritinib are being studied as maintenance therapy after HSCT for AML with FLT3 mutations [7, 10, 56]. FLT3 inhibitors including gilteritinib may have the potential for AML maintenance therapy, though definitive data from clinical trials are not available yet (Table 1).

## Conclusion

Gilteritinib has been approved for R/R AML with FLT3 mutations. The ADMIRAL study showed that longer overall survival and higher response rate are associated with gilteritinib in comparison with salvage chemotherapy for R/R AML. These data from the ADMIRAL study may lead to the therapy paradigm shift and establish gilteritinib as the new standard therapy for R/R FLT3-mutated AML.

## Data Availability

The material supporting the conclusion of this review has been included within the article.

## Abbreviations

CR: Complete remission
CR/CRh: Complete remission with partial hematologic recovery
GCSF: Idarubicin
MEC: Mitoxantrone, etoposide, cyclophosphamide FLAG-IDA Fludarabine, cytarabine
MRD: Minimal residual disease
ORR: Overall response rate
OS: Overall survival
PFS: Progression free survival
