# Correction to: Gilteritinib: a novel FLT3 inhibitor for acute myeloid leukemia

**DOI:** 10.1186/s40364-019-0172-0

**Published:** 2019-10-17

**Authors:** Juanjuan Zhao, Yongping Song, Delong Liu

**Affiliations:** 1 Department of Oncology, The first Affiliated Hospital of Zhengzhou University, Zhengzhou, China; 2 Division of Hematology and Oncology, New York Medical College, Valhalla, USA

**Correction to: Biomark Res (2019) 7:19**

**https://doi.org/10.1186/s40364-019-0170-2**

The original article [1] contains an error in the legend of Fig. 1 whereby it is incorrectly stated that MEC stands for ‘mitoxantrone, etoposide, cyclophosphamide’.

It should instead be stated that MEC stands for ‘mitoxantrone, etoposide, cytarabine’.
